# The Critical Role of Cardiac Magnetic Resonance Imaging in Evaluating Patients With Eosinophilic Granulomatosis With Polyangiitis

**DOI:** 10.7759/cureus.10279

**Published:** 2020-09-06

**Authors:** Saijanakan Sridharan, Saruja Nanthakumaran, Manoj R Somagutta, Sukrut Pagad, Ashley A Arnold, Vanessa May, Bilal Haider Malik

**Affiliations:** 1 Research, California Institute of Behavioral Neurosciences & Psychology, Fairfield, USA; 2 Internal Medicine, California Institute of Behavioral Neurosciences & Psychology, Fairfield, USA; 3 Surgery, California Institute of Behavioral Neurosciences & Psychology, Fairfield, USA

**Keywords:** eosinophilic granulomatosis with polyangiitis, cardiac mri, churg-strauss syndrome, vasculitis, rheumatology, internal medicine, internal medicine and rheumatology

## Abstract

Eosinophilic granulomatosis with polyangiitis (EGPA) is a rare autoimmune systemic necrotizing vasculitis of blood vessels that often presents with hypereosinophilia. Cardiac involvement in EGPA directly correlates with the mortality of patients with the disease and is a central part of the disease process. The evaluation and treatment of cardiac anomalies are vital in patients with EGPA. The frequency with which cardiac involvement is seen in the disease process makes early diagnosis crucial in all patients with EGPA. Early treatment has been proven to reverse or cause the disease to go into remission. Several studies have shown that cardiac magnetic resonance (CMR) imaging is the most sensitive and best early indicator of cardiovascular involvement in EGPA. CMR routinely outperforms other diagnostic techniques such as ECG (echocardiography) and CTA (computed tomography angiography) in the detection of cardiac anomalies and should be a part of the standardized assessment of all patients with EGPA. CMR is also a non-invasive diagnostic tool that can also outperform biopsy in the detection of EGPA cardiac involvement. CMR is also a valuable technique that can be used to monitor disease progression while treatment is being performed. Although long-term research studies have yet to show these benefits, the studies that are available today provide ample evidence that shows CMR imaging could ultimately help bring down mortality rates currently seen in EGPA patients if it is used as an evaluation tool from initial diagnosis and throughout the entire course of disease management.

## Introduction and background

Eosinophilic granulomatosis with polyangiitis (EGPA), formerly Churg-Straus syndrome, is an autoimmune systemic necrotizing vasculitis of small- to medium-sized blood vessels that presents with hypereosinophilia [1-3]. Cardiac involvement in EGPA is found in a clinically significant amount of patients [4]. The spectrum of cardiac deficits includes myocarditis, coronary vasculitis, valvular heart disease, pericarditis, and rhythm disorders [5,6]. Untreated cardiac deficits are associated with increased mortality and poor prognosis. CMR, fluorodeoxyglucose positron emission tomography (FDG-PET) scan, echocardiography (ECG), and endomyocardial biopsy (EMB) are standard methods of detection of cardiac pathology [5,7-10]. ECG is the only technique part of the regular protocol currently used in EGPA patient management.

The early detection of cardiac involvement in EGPA is crucial due to the poor prognosis and increased mortality seen in these patients [4,5]. The current guidelines outline the importance of ECG for the detection of heart failure; however, the use of CMR, FDG-PET, ECG, and EMB synergistically can allow for the early detection of extensive cardiac involvement seen in EGPA. Evaluating patients with ECG helps systematically screen for heart failure, neglecting the early involvement of the cardiovascular system, which is often detected by CMR. CMR is the only non-invasive diagnostic tool that can reliably produce information on early cardiovascular involvement in patients with EGPA. CMR also provides high spatial resolution, which permits thorough evaluation of morphology, function, and tissue status all at the same time [11]. CMR imaging can reveal late gadolinium enhancement (LGE), indicating early fibrosis and coronary vessel involvement that ECG fails to show.

Quantifying and evaluating risk based on cardiac involvement is paramount to the successful treatment of EGPA patients, leading to decreased cardiac mortality rates. The emergence of a non-invasive imaging technique, such as CMR, can provide adequate information that allows medical professionals to make early decisions on treatment and prevention, which could decrease mortality [12]. This study aims to demonstrate the importance of extensive cardiac imaging, primarily CMR, as a central part of the routine diagnostic workup and evaluation of the progression of cardiac anomalies in EGPA patients.

## Review

Literature was searched in PubMed using a combined strategy of Medical Subject Headings (MeSH) and regular keywords for the collection of data. Keywords included EGPA, cardiac MRI, myocarditis, eosinophilia, vasculitis, and Churg-Strauss Syndrome. Only articles written in the last 15 years were included in the study. As a result of the various combinations of keywords, MeSH, and inclusion criteria, 989 total articles were found to be potentially relevant to the study. Careful screening of the abstracts of these articles resulted in 57 articles that matched the objective or provided relevant information to this study. Of these 57 articles, 28 were ultimately used in the study as they provided necessary information that was essential to this review.

Cardiac involvement 

Cardiac involvement in EGPA has been well established and credited with greater significance in disease outcomes when compared to other organ systems. The frequency with which the cardiovascular system is involved in the disease process of EGPA is alarmingly high, reaching levels as high as 92% [13]. The importance of accurate and reliable detection of this involvement is especially critical due to the high frequency of cardiac involvement in the EGPA population, and the high degree of mortality that comes with it. In addition, a study by Fijolek et al. showed that 100% of the 32 patients enrolled presented with myocardial injury [14]. A study by Szczeklik et al. also showed cardiac involvement in 18/19 patients through varied diagnostic techniques such as ECG and CMR [13]. As a result of the alarmingly high frequency with which cardiovascular involvement is found, the diagnostic approach should reflect this degree of involvement in EGPA. Detection and subsequent treatment can be used to combat the high mortality rate seen with cardiac involvement.

Importance of early treatment

The benefits of early treatment in patients with cardiovascular issues in EGPA are the driving force behind the importance of early detection. Several studies have shown that the use of standard therapies early in disease often provides the best outcomes [15,16]. Early and aggressive treatment using corticosteroids and cyclophosphamide have consistently proven to improve cardiac outcomes as well as the global outlook for EGPA patients [16]. A study by Jeong et al. has shown that the cardiac involvement in EGPA can often be due to dilated cardiomyopathy (DCM), which is often reversible. Aggressive treatment in this particular DCM patient showed improvement within three months of symptom detection and treatment initiation [17]. A study by McAleavey et al. also showed the case of a patient who presented with acute myocarditis as shown by CMR, who was subsequently put on a course of methylprednisolone and cyclophosphamide. This patient subsequently showed no persistent myocardial changes or any persistence in gadolinium enhancement [16]. These two studies showed rather quick clinical improvement due to the early treatment, which was made possible by early detection. A study by Neumann et al. followed 42 patients with cardiac involvement, who were subsequently treated with high-dose oral corticosteroids and various immunosuppressive agents. All the patients in this study achieved early remission [15]. The early treatment of EGPA-induced cardiovascular disease plays a significant role in symptom reversal and the achievement of early remission, highlighting the importance of early detection.

CMR use in diagnostics

The complexity behind the diagnosis of EGPA provides unique challenges, and the involvement of the cardiovascular system in the disease process can often be overlooked. It is important to remember the high mortality rate associated with cardiovascular involvement in EGPA, and therefore prioritizing the evaluation of these issues is paramount [18,19]. The diagnosis of EGPA itself has varying challenges, one of which is the fact that a patient with cardiac involvement may present with anti-neutrophil cytoplasmic antibody (ANCA) positivity less frequently than a patient without cardiac involvement [16]. The current use of ECG for the detection of cardiovascular involvement is limited to evaluating left ventricular (LV) wall motion and functional changes [20]. The shortcomings of ECG include the inability to identify tissue damage and the detection of other types of damage before there are apparent LV abnormalities [20]. The absence of chest pain and ECG abnormalities can lead to a false sense of security, even in patients with increased troponin levels. CMR may also be a better tool to evaluate cardiac involvement than EMB. CMR is non-invasive and is not limited to providing information about only some portions of the heart, which is often a limitation with EMB [17].

CMR can reveal edema and persistence of gadolinium enhancement, which can indicate myocardial involvement and scar tissue formation [16,21]. In patients with decreased ejection fraction (EF), <30%, on ECG and a normal coronary angiogram with limitations against EMB, CMR can prove to be an important diagnostic tool that allows for the detection of subendocardial involvement through LGE [22]. The unique challenges that are present in making the diagnosis of EGPA are only further compounded by the different sets of challenges in evaluating cardiac involvement at the same time. The involvement of the cardiovascular system in EGPA is detected with great accuracy through the use of CMR. CMR is also able to reliably relay information in patients who may be medically unable to undergo specific diagnostic procedures such as EMB.

CMR performance compared to other imaging modalities

The evaluation of the cardiovascular system in EGPA patients involves the use of many imaging techniques including ECG, computed tomography angiography (CTA), and CMR [23]. The EGPA Consensus Task Force has stated that the use of advanced imaging techniques showed more sensitivity in detecting cardiac involvement [24]. ECG has reliably shown its ability to detect the presence of heart failure in EGPA, with its main limitation being that it shows damage after it has already been inflicted [20]. CMR has consistently outperformed ECG in the early detection of cardiovascular system involvement in EGPA [25,26]. A study by Dennert et al. indicated that ECG showed cardiac abnormalities in 50% of EGPA patients, whereas the same group showed various abnormalities in the cardiovascular system when evaluated with CMR. The abnormalities detected included wall motion abnormalities, focal fibrosis, and obliteration of the right ventricle [25]. The same study showed that CMR detected cardiac abnormalities with higher sensitivity than ECG (88% versus 82%) [25]. A study by Dalia et al. also indicated CMR's capability when a patient with cardiovascular involvement in EGPA exhibited apical hypokinesis with an EF of 55% [26]. However, upon evaluation with CMR, there were several areas of delayed enhancement in the LV myocardium, and decreased perfusion in mid to apical septal and inferior segments through the cardiac apex [26]. The study conclusively demonstrated the strengths of CMR use as part of a new strategy to treat these patients before the progression of damage becomes insurmountable. Also, the patient in that study did not show any areas of stenosis, calcified plaque, or soft plaque upon evaluation with CTA [26]. The use of CMR has proven to be the most effective imaging technique and diagnostic tool for evaluating cardiovascular disease in EGPA patients. Studies have shown that it reveals cardiac involvement in EGPA with greater sensitivity versus other commonly used imaging techniques such as ECG or CTA. The findings of the various studies show the superiority of CMR as an imaging technique and should lead to the use of CMR with greater frequency and urgency.

CMR utilization and disease progression

EGPA patients face challenges with cardiovascular involvement, which can often go unmonitored or even unchecked. While CMR has established itself as a reliable diagnostic indicator of cardiac involvement, it can also be used to provide snapshots over a period of time to help monitor cardiovascular disease progression or remission. In a study by Dunogué et al., the detection of cardiomyopathy in EGPA patients was assessed using LGE. Fifteen patients with cardiomyopathy detected through LGE were then reassessed through CMR after receiving the appropriate treatment. CMR detected an improvement in seven patients while detecting stabilization or worsening of the remaining eight patients [27]. In a study by Marmursztejn et al., 14 out of 20 patients who were classified as being in remission from EGPA showed signs of myocardial involvement when evaluated through CMR. Of the 14 patients, two patients showed signs of acute inflammation on FDG-PET, indicating that active myocardial inflammation can persist in patients who are considered to be in remission [28]. A study by Fijolek et al. showed that 10 of 12 patients in clinical remission after undergoing treatment for EGPA still presented with signs of change on CMR imaging [14]. Studies have shown the ability of CMR to evaluate cardiovascular involvement throughout the entire disease progression of EGPA. CMR can be used effectively to determine any improvement, stabilization, or worsening of cardiovascular disease in these patients. Even after remission is achieved clinically, the use of CMR can help in the detection of lingering cardiac issues that persist even after clinical symptoms may have diminished. CMR has also been useful in detecting clinical improvement in patients using standard treatments. The use of CMR for detection and evaluation throughout the various stages of disease has shown the versatility and reliability of CMR as a diagnostic tool.

## Conclusions

The involvement of the cardiovascular system in EGPA is the most important prognostic indicator, which is directly correlated to the mortality rate seen with this disease. The emergence of several imaging techniques has been especially beneficial in the early detection and subsequent treatment in the progression of this disease, which should eventually result in a better outcome for all patients. CMR imaging has proven to be the most sensitive diagnostic technique consistently across many studies. It also has the added benefit of being a non-invasive tool, which provides greater patient comfort with the potential for fewer complications. The limits of the various diagnostic techniques currently employed have been clearly outlined in many studies and in practical use, especially in the context of early and non-invasive detection. The use of CMR should be implemented early in the course of the disease and should also be used to monitor any further progression or regression of disease effectively while using standard treatment options. The high prevalence of cardiac anomalies in EGPA only serves to reiterate the importance of these conclusions. CMR outperforms many other diagnostic tools that are currently used as the standard method of evaluation. An approach that would utilize the many different diagnostic techniques, while employing CMR as the central part of the routine diagnostic workup in the evaluation of cardiac anomalies, would lead to the best outcomes for all patients with EGPA.

This study acknowledges the lack of published long-term research to establish a truly comprehensive look at the long-term outcomes of patients with EGPA and how the use of CMR will provide better clinical results in these scenarios. This study concludes the significance of CMR and how it can be best utilized as a diagnostic evaluation tool throughout the course of the disease. CMR should be a primary part of the routine evaluation process to help decrease mortality rates in patients with EGPA.
